# Assessing the health impacts of transnational corporations: a case study of Carlton and United Breweries in Australia

**DOI:** 10.1186/s12992-022-00870-0

**Published:** 2022-09-09

**Authors:** Julia Anaf, Fran Baum, Matt Fisher, Fiona Haigh, Emma Miller, Hailay Gesesew, Nicholas Freudenberg

**Affiliations:** 1 Stretton Health Equity, Stretton Institute, North Tce Campus, University of Adelaide, Adelaide, 5005 Australia; 2 Health Equity Research Development Unit *HERDU, UNSW Centre for Primary Health Care and Equity (CPHCE), University of New South Wales, Sydney, 2052 Australia; 3 Clinical Services Integration and Population Health, Sydney Local Health District, Sydney, Australia; 4 College of Medicine and Public Health, Flinders University of South Australia, GPO Box 2100, Adelaide, South Australia 5001; 5 Torrens University Australia, 88 Wakefield St, Adelaide, South Australia 5000; 6 Graduate School of Public Health and Health Policy, City University of New York, New York, USA

**Keywords:** Alcohol industry, Globalization, Health equity, Transnational corporations

## Abstract

**Background:**

The practices of transnational corporations (TNCs) affect population health through unhealthy products, shaping social determinants of health, or influencing the regulatory structures governing their activities. There has been limited research on community exposures to TNC policies and practices. The aim of this paper was to adapt existing Health Impact Assessment methods that were previously used for both a fast food and an extractives industry corporation in order to assess Carlton and United Breweries (CUB) operations within Australia. CUB is an Australian alcohol company owned by a large transnational corporation Asahi Group Holdings. Data identifying potential impacts were sourced through document analysis, including corporate literature; media analysis, and 12 semi-structured interviews. The data were mapped against a corporate health impact assessment framework which included CUB’s political and business practices; products and marketing; workforce, social, environmental and economic conditions; and consumers’ adverse health impacts. We also conducted an ecological study for estimating alcohol attributable fractions and burdens of death due to congestive heart disease, diabetes mellitus, stroke, breast cancer, bowel cancer and injury in Australia. Beer attributable fractions and deaths and CUB’s share were also estimated.

**Results:**

We found both positive and adverse findings of the corporation’s operations across all domains. CUB engage in a range of business practices which benefit the community, including sustainability goals and corporate philanthropy, but also negative aspects including from taxation arrangements, marketing practices, and political donations and lobbying which are enabled by a neoliberal regulatory environment. We found adverse health impacts including from fetal alcohol spectrum disorder and violence and aggression which disproportionately affect Indigenous and other disadvantaged populations.

**Conclusion:**

Our research indicates that studying a TNC in a rapidly changing global financialised capitalist economy in a world which is increasingly being managed by TNCs poses methodological and conceptual challenges. It highlights the need and opportunity for future research. The different methods revealed sufficient information to recognise that strong regulatory frameworks are needed to help to avoid or to mediate negative health impacts.

**Supplementary Information:**

The online version contains supplementary material available at 10.1186/s12992-022-00870-0.

## Background

Extensive literature examines negative impacts from the products and operations of the alcohol industry [1–4]. These impacts begin with the brewing process, as the grain, glass, and product delivery all leave environmental footprints. Grain farming and beer production involve high water consumption and waste generation, while transportation and retail refrigeration are energy intensive [5, 6]. It is estimated that alcoholic beverages, including beer, account for 0.7% of global greenhouse gas (GHG) emissions across the complete product lifecycle [7].

In Australia, more than 5500 lives are lost, and 157,000 people are hospitalised annually as a result of alcohol consumption [8]. Negative individual and societal impacts involve loss of life, including from suicide [9], increased disease risk, crime, and road accidents [10]. These together with healthcare costs including hospitalisation, labour costs, and child protection services for dealing with child maltreatment, are estimated to cost Australia Au$36 billion annually [10]. Alcohol is estimated to be a factor in up to 65% of reported family violence incidents and in up to 47% of child abuse cases each year in Australia [11]. The experience of family violence can lead to the use of alcohol consumption as a coping mechanism, and children who witnesses violence, or its threat between parents, are more likely to display harmful drinking patterns later in life [12].

The alcohol industry also contributes to chronic health conditions [13]. Alcohol is a risk factor for cardiovascular disease, several kinds of cancer, chronic liver and pancreatic diseases, and mental illnesses [13, 14]. According to the World Health Organization [15] the harmful use of alcohol accounts for 7.1 and 2.2% of the global burden of disease for males and females respectively. It is the leading risk factor for premature mortality and disability among people aged 15 to 49 years. Disadvantaged and particularly vulnerable populations have higher rates of alcohol-related death and hospitalisation [15]. Alcohol consumption in pregnancy can result in fetal alcohol spectrum disorder (FASD) and other adverse health effects for the child including brain damage, congenital anomalies, and cognitive, emotional, behavioural, and adaptive functioning deficits [16, 17].

In Australia, alcohol consumption is skewed towards heavy drinkers, with the top 10% of heaviest drinkers consuming more than half of all alcohol [18]. Most alcohol in Australia is marketed and distributed through a small number of transnational corporations (TNCs) and national companies [19, 20]. The global alcohol industry comprises producers, distributors, retailers, marketers and social aspects organizations such as DrinkWise in Australia that create an impression of social responsibility but promote interventions that further the interests of the alcohol industry [21, 22]. These diverse, high-level industry links lead to enormous global financial and political power which can undermine public policies to regulate the alcohol industry in the interests of public health and wellbeing [23].

There is a growing body of literature examining health impacts of the corporate alcohol industry. This includes industry influence over trade agreement negotiations (e.g. with the World Trade Organisation) to promote industry interests ahead of public health [24, 25]. Alcohol control is also a contested policy field, with different framing by industry and public health actors with the aim of influencing bodies such as the World Health Organisation [26] globally, and national governments.

The alcohol sector and individual alcohol TNCs and retail outlets therefore act as commercial determinants of health (CDoH), or the strategies and approaches employed by the private sector to promote products and choices that are detrimental to health [27, 28]. Although extensive research has been conducted on the health impacts of alcohol, less has focused on the alcohol industry, and very few researchers have studied individual alcohol TNCs [19]. This paper adds to the knowledge base by helping to address a research gap highlighted in the public health literature. This is that a focus on individual industry sectors alone does not address the individual corporation as ‘a foundational societal institution that affects health’ [29 p.6]. Documenting both positive and negative impacts of individual TNCs can help to identify the types of changes that a corporation can implement in order to optimise public health, or at least be less damaging [30]. Information from individual Corporate Health Impact Assessments (CHIAs) may also enable health advocates to operate from a stronger evidence base, and inform governments of the potential need for policy responses [30].

This study of the health impacts of one Australian alcohol company, Carlton and United Breweries (CUB) (a wholly-owned subsidiary of Japanese-based Asahi Group Holdings (Asahi) is the first to apply health impact assessment to such a TNC.

This paper provides a multi-pronged analysis supported by an overarching framework which captures health impacts of CUB. These impacts relate to the broader regulatory framework, the company’s political and business practices, products, distribution and marketing, and impacts on daily living conditions.

These living conditions include workforce, social and economic conditions, impacts on the natural environment, as well as adverse health impacts from consumption and associated costs. An ecological study also estimates attributable fractions and burdens of death from chronic illnesses and injury, including CUB’s share.

## Methods

### Health impact assessment

Health Impact Assessment (HIA) is a structured, evidence-based and solution-focused process for predicting future health consequences, and for maximising positive and minimising negative health impacts of policy, plans, projects, or programs [31, 32]. Health impact assessment can be carried out prospectively at the beginning of a planned activity, concurrently taking place while the proposal is being implemented, or used retrospectively as an evaluation tool [33]. It is most often used prospectively so that the findings can inform and strengthen planning processes. Health impact assessment can be a mechanism for developing relationships with, and influencing other sectors [34].

The formal HIA process incorporates six steps: screening, scoping, identification, assessment, decision-making and recommendations, and evaluation and follow-up [31]. Health impact assessments can be carried out at different depths depending on area of focus, timing, and available resources. They can range from desktop level (that may take a few hours or days) to comprehensive (involving many months, a wide range of areas of focus and primary data gathering). This is an intermediate HIA carried out over 12 months and involving a mix of secondary and primary data collection.

We adapted existing HIA methods to focus on TNCs, guided by a Corporate Health Impact Assessment (CHIA) framework [35] which we have employed in our prior research on TNCs in the food and extractive industry sectors [36, 37]. This framework was developed in 2016 in collaboration with academics, civil society actors and the corporate sector [35].

A CHIA broadly follows typical HIA steps and processes but does not include the formal monitoring and evaluation stages. A CHIA differs from standard HIA practice in that it looks at an industry or company rather than a proposal. This CHIA is a concurrent HIA, examining the existing health and health equity impacts of a corporation with a view to informing and influencing future practice. Identifying both positive and negative health impacts may provide insights for TNCs to improve their operations and reputational standing, governments to develop appropriate regulatory frameworks, and civil society groups to organize campaigns to strengthen a company’s positive and reduce its negative outcomes. The CHIA framework is reproduced in the Results section. A group of seven academics with particular experience in HIA, mixed methodologies, and CDoH led the project. See Table 1.

**Table 1:** Documenting the CHIA process

Step	Objective	What we did	Outcomes
Screening	To decide whether a HIA is feasible, timely, and would add value to the decision making process	The CHIA team identified Alcohol industry as suitable focus for further development of CHIA methodology. CUB identified as suitable candidate for Alcohol company operating in Australia	Decision made to conduct a CHIA to inform development of CHIA methodology and also to provide evidence and recommendations to influence CUB practice in relation to CDoH.
Scoping	To create a plan and timeline for conducting a HIA that defines priority issues, research questions and methods, and participant roles	Scoping meeting held with the working group to determine focus of assessment.	Identified: Focus areas: Estimated health impacts including CUB’s share of alcohol attributable fractions and death burden. Focus populations: Australian population Focus timeframes: Current Geographic focus: Australia
Identification	Collect evidence to identify potential health impacts	Evidence compiled included: • Corporate literature • Media reports • Semi-structured interviews with experts and key stakeholders • Ecological analysis	Evidence summaries developed.
Assessments	Synthesise and critically assess the information in order to prioritise health impacts: To provide evidence based recommendations to mitigate negative and maximize positive health impacts. Make decisions to reach a set of final recommendations for acting on the HIA’s findings.	A workshop with working group to discuss and validate the findings of the assessment and develop recommendations for policy options and response.	CHIA framework populated Draft recommendations. Impact characterization.
Report on health impacts and recommendations	To develop the HIA report and communicate findings and recommendations	Findings compiled by the CHIA team.	A paper for peer reviewed publication developed
Monitoring and evaluation	Monitor and collate responses to HIA publication		Use findings from monitoring response to inform further development of framework and future application

### Screening: selecting the corporation for the study

Criteria for choosing which corporate sectors and particular TNCs to assess in a CHIA include consideration of the attributable burden of disease and the wider socioeconomic context under which a company operates [36]. We selected CUB for a case study of an Australian alcohol corporation to extend our work on the health impacts of individual TNCs in other industry sectors [36, 37]. We have not undertaken any prior research on CUB. The criteria for selecting CUB included that it is a major company in the sector; with business and political practices that potentially impact significantly on health, especially that of vulnerable populations; and with a public image and brand recognition that are familiar to the general population in Australia. Although we considered aspects of its parent company Asahi’s role as owner of CUB in our CHIA, the project scope did not extend our analysis to Asahi’s broader global operations.

### Identification of potential impacts: data collection

We sought data highlighting both positive and adverse health impacts related to CUB products and operations by qualitative analysis of a range of documents including corporate literature, media items, semi-structured interviews, and by undertaking a quantitative ecological analysis. We took an iterative approach for qualitative data collection, preliminary analysis, and additional data collection [38]. This was to allow us to accrue any new information as the study progressed [39].

#### Documents

Selection criteria for the initial document collection were currency (from 2016 onwards) and relevance for informing the scope of domains spanning the CHIA framework. We accessed the Flinders University library holdings from 2016 using the search term ‘Carlton and United Breweries’ in the Title field and limited to English. This returned information from all subscription databases, all physical holdings, and all open access journals. Of the 145 items retrieved only 14 dated from 2016 or later. The Google Scholar database was accessed using the same search term and timeframe but returned very limited information. All alcohol-related items in the Endnote library compiled to inform the project were checked for specific mention of CUB or Asahi. After review, 51 documents were initially retained for analysis. We undertook later purposive and opportunistic web searches to add pertinent information as the project developed, including internet searches for any new and relevant documentation of CUB and Asahi.

We accessed the Asahi and CUB websites for corporate literature including corporate social responsibility and sustainability statements, information on products and marketing, and any submissions to government. We checked Hansard records for references to CUB. Internet searches were also conducted for information on peak alcohol and other industry bodies affiliated with CUB.

#### Media items

To help identify media reporting on CUB, we consulted the Factiva database from 2016 onwards using the broad search term ‘Carlton and United Breweries’ for any references relevant to the CHIA framework. Of the 438 items retrieved 197 were duplicates, with other items perused for potential relevance. The Proquest ANZ Newsstream database was also accessed for Australian mainstream media using the same search term and timeframe; identifying 15 items. After scrutiny, 38 media items from across both databases were retained for analysis, and supplemented in subsequent targeted web searches undertaken to identify later relevant items.

#### Semi-structured interviews

We interviewed four academics, six civil society actors, and three policy actors for approximately 1 h, with a timeframe according to participant availability and spanning 29 minutes and 75 minutes (See Table 2). Interview participants were selected by purposive and snowball sampling to elicit broad perspectives on CUB’s products and operations. All potential respondents were emailed a personalised invitation, a Participant Information Sheet and a Consent Form. Tailored interview schedules were designed to gain diverse responses from CUB or industry representatives, civil society actors, academics, and policy actors. We selected respondents based on their expertise on the nature and practices of the alcohol industry in Australia and the current government regulation of the industry.

**Table 2:** Interview respondents

Name of group	Number of participants	Source of participants
Academics	4	Academics from four different Australian universities or academic institutions with expertise in alcohol research
Civil society actors	6	Civil society actors from several Australian alcohol advocacy groups
Policy actors	3	Members of Parliament or other key policy actors from different political parties who have acted upon, or expressed interest in the impact of alcohol on population health.

Permission was sought from CUB management to interview several senior executives who could provide an industry perspective, but participation was denied by the chief executive officer. Invitations were sent to eight other alcohol industry or related business representatives, with follow-up invitations sent after 14 days. Four of the potential participants did not respond, and four declined our invitations.

Interviews were digitally recorded and transcribed by professional transcription services. Ethics approval to conduct the study was obtained from the Flinders University Social and Behavioural Research Ethics Committee (Project No. 8663).

#### Estimate of attributable alcohol risk

We conducted an ecological study for estimating alcohol attributable fractions and burdens of death due to congestive heart diseases, diabetes mellitus, stroke, breast cancer, bowel cancer and injury in Australia. Beer attributable fractions and deaths, and CUB’s share for the above-mentioned chronic illnesses and injury were also estimated.

The indicators used in the ecological study included: age standardised mortality, prevalence of drinking (PR_d_), alcohol (and beer) consumption in volume of pure alcohol, beer per capita consumption of pure alcohol, CUB’s beer share, and relative risk (RR) of alcohol consumption for the abovementioned chronic illnesses and injury.

Our analyses included age standardised mortality per 100,000 population from the Australian Institute of Health and Welfare (AIHW) [40]. The sources of PR_d_ for 1990, 2000 and 2005 for Australia were sourced from the 2016 systematic analysis for the Global Burden of Disease Study [41] for; 1998, 2001, 2004, 2007, 2010, 2013 and 2016 from Australia’s National Drug Strategy Household Survey; and for 2008, 2012 and 2014 from Australian Bureau of Statistics (ABS) [42]. We applied mean imputation for the years 1991–1997, 1999, 2003, 2006, 2009, 2011 and 2015. Data for PR_d_ are presented in Supplementary Table 1. The alcohol (and beer) consumption in volume of pure alcohol in ‘000 s of litres and per capita consumption of pure alcohol were obtained from the ABS [42]. Data for CUB’s market beer share were obtained from *IBISWorld* data [43], however data were only available from 2010 onwards.

We searched meta-analytic studies from PubMed and relevant bibliographies to find the RR using the following search concepts: (i) alcohol consumption, (ii) CHD, DM, stroke, breast cancer, bowel cancer and injury, and (iii) systematic review/meta-analysis. Given the RR are different for different levels of alcohol consumption, we considered the RR for Australia’s average consumption level as the cut-off. Average consumption for Australia is reported to be 2.72 standard drinks, or 27.2 g of alcohol per day [44]. Thus, the RRs for 27.2 g per day consumption of alcohol were estimated for CHD, [45] DM, [46] stroke, [47] breast cancer, [48] bowel cancer [49] and injury [50] were 0.8, 0.36, 1.04, 1.14, 1.2, and 13 respectively.

##### Attributable burden due to alcohol use

We calculated PAF using the following formula.

1 $$PAF=\frac{Prevalence\ of\ drinking\ \boldsymbol{X}\ \left( RR-1\right)}{Prevalence\ of\ drinking\ \boldsymbol{X}\ \left( RR-1\right)+1}$$

PAF of 1 (100%) is assumed as fully attributable. Negative PAF values indicate that alcohol is a protective factor. Following the PAF calculation, PAFs were multiplied by outcome-specific age standardised estimates of deaths to calculate the total attributable burden for each outcome in each specific period. To calculate the beer attributable burden for each outcome, we multiplied the total alcohol attributable outcome specific burden by the proportion of volume of alcohol in beer per capita. To quantify CUB’s contribution, we multiplied the beer attributable burden by CUB’s beer share proportion.

### Assessment of impacts

The documents, media items, and transcribed interviews were imported into NVivo qualitative data analysis software and coded against a coding frame that mirrored the CHIA framework (see Table3). The iterative approach to data collection and preliminary analysis allowed for a reflexive process for developing meaning from the data.

## Results

Our results reflect the Identification stage of the CHIA and are presented under the three levels of the CHIA framework. Level A explores the political, economic and regulatory context of CUB’s operations. Level B examines CUB’s practices and products that impact on health and equity. Level C considers the direct impacts of the company’s practices on daily living conditions across five domains: workforce and working conditions, social conditions, natural environment, adverse health impacts, and economic conditions.

Table 3 summarises the data coded against the three-level CHIA framework. Node summaries were compiled according to the main research focus which was to identify the positive and negative aspects of the TNC’s operations populating each subsection across the three CHIA levels. The type of data source (documents including corporate literature, media items, or interviews) was noted against each individual summary listing to assist with the analysis. The summaries were discussed in team meetings and informed the compilation of Table 3 with its notations highlighting potential health impacts, their likelihood, and level of significance.

**Table 3:** Corporate Health Impact Assessment Framework

**A:** ***TNCs and regulation***				
**Global/Regional:** Regulatory environment; Political & economic environment; International institutions *Successive, strategic corporate restructures, sales and acquisitions.* *Limited number of TNCs dominating market/market concentration - commercial power and increased resources for marketing and lobbying. (−)* *Lack transparency – difficulty in monitoring activities (−)* *Corporate consumption complex*	**National:** Regulatory environmental; Political & economic environment; Status & capabilities of national government; Social & economic inequalities *Neoliberal regulatory environment – limited regulation and prioritisation of private interests over population health and equity (−)* *Strong trade associations (ABA, AHA) and lobbying Group (BAA) (−)* *Voluntary Codes of Conduct and limited regulation lacks both independent review and monitoring and consequential sanctions for non-compliance (−)*		**Sub-national/Local**: Regulatory environment; Regions or population groups especially affected by TNC. *Powerful influence on local level licensing (−)*	
**B:** ***TNCs: Health and equity impacts***				
**Structure:** Global and national operational structure and size of TNC; supply chain				
**Political practices:** Actions to influence: Global regulatory or political environment; National regulatory or political environment; Role of industry bodies; Taxation structures; Media Regulatory capture through lobbying and close industry political relationships (−*##LT) ABA advocates for policies and regulations that target specific at-risk groups rather than broader population-based initiatives (−**##LT) AHA – track record opposing public health measures (−***###LT)	**Business practices:** Control over supply chain; Labour practices; Taxation payments/profit shifting; Use of litigation; Use of trade/investment treaties to influence national regulations Multiple ownership and concentration of power changes (−*##LT) Sustainability goals (+*#LT)		**Products, distribution and marketing:** Product types, trends, proportions; Export vs domestic sale; Marketing methods & strategies Sponsorship of sporting codes provides financial support to sports encouraging physical activity but creates positive link between sport and alcohol consumption (+, −**##LT) Marketing of low alcohol potentially targeting young people (−**##LT) Marketing promoting drinking culture/family friendly (−***##LT) Opposition to pregnancy labelling and other warnings (−**##LT)	
**C:** ***TNC impacts on life domains***				
**Workforce & work conditions** Workforce; Wages; Health & safety; Living conditions Investment in sustainable operations (+* LT##) Employment opportunities (+**I,LT) equal pay and inclusive parental leave (+*I,LT##) Work heath and safety standard (+**I,LT##) Senate enquiry avoidance fair work act (−*,I)	**Social conditions** Local goods & services; Local community life Philanthropic activities benefiting communities (+*I#) Social benefits of alcohol consumption (+**I#) Negative impacts on community life (violence, alcohol related harm) (−***I,LT###) Police, ambulance, hospital, and alcohol treatment services, and child protection systems (− ***I,LT ###) Positive relationship between alcohol and gambling (− ***I,LT ##)	**Natural environment** Ecological systems; Land, water, GHG emissions, pollutants Commitment to reducing environmental footprint (+ * LT#) High water useage in production (−*I,LT##)	**Adverse health impacts** Food consumption; Cost of goods 45% share of alcohol consumption and related direct and indirect impacts (− ***I,LT###) Disproportionate impacts (− **I,LT###)	**Economic conditions** Impacts on national or local economy; Public revenue; Local production systems Employs 1700 staff (+**I#) Tax contribution (+**I#) However externalised industry costs (health, lost productivity etc) (−***I,LT###)
Assess potential health impacts from TNCs activities on: area of impact, type of impact (+ve, −ve), populations affected, size of impact, likelihood (if prospective), timing (urgency) is this fair or avoidable? Likelihood Possible * May happen. Plausible, but with limited evidence to support Probable ** More likely to happen than not. Direct evidence from limited sources Definite ***-Strong direct evidence from multiple sources **Health and Health Equity Impact significance** **Minor # – minimal impact** **Moderate ## – Some impact** **Major ###– very significant** **Timing** **Immediate - ILong term -LT**				
**D. Recommendations** Recommendations for legislation / policy / practice including who is responsible for taking action, the likelihood that action can be taken, timeframes for taking action				

### CHIA level A: how regulatory structures impact on TNCs

Corporate Health Impact Assessment level A captures information related to the global regulatory environment, the political and economic environment, and international institutions. The data included global level information on CUB’s parent body Asahi’s codes of conduct, sustainability principles, and compliance with regulations, including taxation.

### CHIA level B: practices and products that impact on health and equity

In this section we discuss CUB’s corporate structure, political and business practices, and products, distribution, and marketing. CUB was established in 1907 and produces beer, cider, mixed drinks and spirits. It employs approximately 1700 staff across breweries, offices and distribution centres, with Au$1.54 billion revenue raised in 2019 [51]. Over the last decade the rapidly changing ownership between major global alcohol TNCs has affected CUB. Between 2011 and 2019 CUB had four changes of ownership. In 2019 CUB was acquired by the Japanese TNC Asahi Group Holdings for Au$16 billion.

#### CUB’s political practices

Carlton and United Breweries’ political practices include actions which influence the regulatory or political environment, and highlights the role of affiliated industry bodies, within Australia. CUB, together with the brewers Coopers and Lion, account for 80% of Australian beer production and sales. These brewers comprise the three members of the peak alcohol body, Brewers Association of Australia, of which CUB’s CEO, Peter Filipovic, is the chair. The Brewers Association lobbies government on behalf of its members, and is affiliated with a range of other peak industry bodies which ‘play a role in influencing the policy and reputation of the sector’ [52]. These bodies include Alcohol Beverages Australia (ABA), the national body representing the interests of alcohol beverages manufacturers, distributors, retailers and drinkers. Both Asahi and CUB hold corporate membership of the Australian Hotels Association (AHA), the peak body representing the interests of employers in the hospitality and liquor industry. The AHA provides a ‘strong platform for influence’, focusing on alcohol and gaming policy, trade practices, workplace relations, taxation, and business regulation [53].

Alcohol marketing is governed by a voluntary code, the Alcohol Beverages Advertising Code (ABAC) scheme for responsible alcohol marketing [54]. Carlton and United Breweries is a signatory, with Brewers Association representatives on the management committee. Although it has a degree of accountability and transparency, the scheme lacks both independent review and monitoring and consequential sanctions for non-compliance; thus undermining its credibility [55].

Carlton and United Breweries is also a member and funder of DrinkWise Australia, a Social Aspects and Public Relations Organisation (SAPRO) and large-scale charity established in 2005; funded by voluntary contributions from the alcohol sector in Australia [56, 57]. When it was first established, it received funding of $5 million from the alcohol industry and $5 million over 4 years from the Australian Government. Since 2009 it has been funded exclusively by alcohol producers, distributors and retailers [57].

Carlton and United Breweries’ links to peak industry bodies also allows it to influence government through direct and indirect lobbying, and through political donations that are often targeted at times of critical policy debates or immediately before elections [58]. The power and influence of lobbying and political donations was raised by several research participants. One stated:

*I think there are lots of decisions made in government that are unduly influenced by industry connections, either through lobbying or donations or the ‘revolving doors’ between boards and governments. (Academic #2)*

Alcohol industry lobbying included opposition to the Food Standards Australia and New Zealand (FSANZ) proposals for stronger mandatory pregnancy warning labels to help reduce the incidence of FASD [59]. According to one respondent:

*I know the Brewers Association was one of the groups that was lobbying hard and lobbying the Minister and met with the Minister early this year to push back on mandatory labelling (for FASD). (Policy actor #3)*

#### CUB’s business practices

Business practices include labour relations, taxation strategies, and use of litigation and trade and investment treaties in ways to influence regulation. Carlton and United Breweries engages in a range of business practices that may benefit the community. Its website reveals sustainability goals including commitment to fuelling local operations with 100% renewable energy by 2025; a commitment matched by its parent body Asahi [60]. Carlton and United Breweries has recently engaged in a range of corporate philanthropy initiatives, including during the COVID 19 pandemic.

Carlton and United Breweries also commits to reducing its environmental footprint through schemes such as circular packaging, going plastic-free or using recycled plastic by 2025, and investing in ‘smart agriculture’ and water stewardship. It supports diversity and inclusion in the workplace, equal pay and inclusive parental leave [61].

However, negative aspects of CUB’s business operations have also been documented. These include taxation practices, opposition to mandatory health warnings, and moves towards labour hire contracting with reduced pay and conditions for workers [62]. It is reported that although parent body Asahi generated $1.7 billion in total revenues in Australia in the 2017–2018 financial year, it paid only $1.2 million in taxation, none in the previous 2 years, and a total of $16.4 million between 2013 and 2015 [63, 64]. Interpreting raw figures and monitoring corporate taxation liability and compliance is complex due to the interplay between international and domestic rules and the capacity for strategic, legal taxation practices [8]. As corporate taxation is paid on profits, changing business structures and ownership affect corporate profitability and thus taxation liability. Carlton and United Breweries’ economic contribution from a range of other taxes is discussed in CHIA level C.

#### CUB’s products, distribution and marketing

The CUB website provides extensive information on its product range; including beers with no, low, medium, and high alcohol content, and different nutritional and energy formulations [65, 66]. Carlton and United Breweries spent Au$12.3 million on media exposure in 2016 [67], and engages in creative marketing strategies to promote products according to customer profiling. For example, zero-alcohol beer is presented with similar packaging, branding and logos as its full-strength beer. While offering choice, and ostensibly being marketed to health conscious consumers, alcohol-free beer has been criticised for potentially targeting young people as future alcohol consumers. Carlton and United Breweries’ website shows imagery suggesting that marketing is mainly directed to young adults through sporting or celebratory contexts [68]. CUB also highlights the importance of digital reach for successful marketing, as revealed in the words of CUB’s Head of Integrated Marketing:

*Experience is not a place; it’s everywhere thanks to digital, and we want our brands to deliver experiences everywhere and anywhere we can play a meaningful role in someone’s life* [69]*.*

One respondent stated that CUB:

*… has been successful in promoting a drinking culture in Australia that is centered around their products; and glorifying that culture has been very successful for their profits but not very successful in terms of the harms that alcohol causes and the way it’s been embedded in Australia society. They are an iconic brand … so they have helped to normalise very high levels of drinking. (Civil society actor #3)*

This respondent also spoke about targeted marketing to Indigenous people:

*If [profit] means marketing to vulnerable people who drink excessively, well so be it. If there’s no regulatory framework to stop them doing that, they will. … It’s quite rational to do that from the point of view of their shareholders. (Civil society actor #3)*

Carlton and United Breweries’ alcohol advertising is monitored under the voluntary ABAC code which provides advertising guidelines and a complaints mechanism [70]. Of the 61complaints received against CUB since 2006, between 1st January 2020 and 1st January 2021, 12 complaints were adjudicated, with four being upheld and eight being dismissed [71]. However, as one respondent maintained:

*Voluntary self-regulation is utterly ineffective. It notionally prohibits targeting of children and young people and that’s complete baloney. It does not prevent the exposure of children and young people to alcohol... The marketing through sport is just everywhere and it’s unconstrained … You have children seeing their idols as walking, running billboards for alcohol. (Academic #1)*

One participant noted that the alcohol industry was marketed as an essential service or an essential good during the pandemic, and that restrictions imposed on liquor retailers were lifted very quickly following industry lobbying (Civil society actor #2). CUB is reported as considering a direct-delivery model for consumers [72].

### CHIA level C: direct impact of CUB practices on daily living conditions

In CHIA level C we highlight the direct impact of CUB’s practices on daily living conditions in five domains: 1) workforce and working conditions, 2) social conditions, 3) the natural environment), 4) health and equity outcomes, and 5) economic conditions. Indicative findings are highlighted below, with additional findings summarised in Table 4.

**Table 4:** Direct impact of TNC practices on daily living conditions

*Work and workforce conditions*	
• CUB’s operational workforce is covered by a range of enterprise agreements negotiated with relevant unions and approved by the Fair Work Commission.	
• CUB has flexible work practices support work-life balance, including job sharing, telecommuting, part-time employment and flexible working hours.	
• CUB provides eight weeks paid maternity leave in addition to government paid parental leave, redundancy pay, paid domestic violence, and family leave.	
• *However*, the alcohol industry, especially the retail sector, is heavily casualised, with implications for employees’ willingness to speak out about health and safety issues.	
• CUB has sought to outsource workers since 2009 through the use of labour hire companies. In 2016, 55 workers were told to reapply for their jobs with severely reduced wages. Hansard records reveal aggressive and threatening comments made by CUB management at the time.	
• In 2020 CUB was investigated by the Fair Work Ombudsman for allegedly inadvertently underpaying Au$1million in penalty rates to 635 hospitality workers over a 10 year period.	
*Social conditions*	
• Social drinking can provide enjoyment, with local hotels, especially in country areas, providing an important meeting place.	
• *However*, there are high levels of alcohol-related violence affecting local communities including street violence, and domestic and family violence.	
• Police, ambulance, hospital, and alcohol treatment services, and child protection systems are all negatively impacted by the industry, with emotional impacts on families.	
• Alcohol dependence is both a health and social issue with addiction harming families and communities.	
• Aboriginal and Torres Strait Islander children comprise many of the young people aged between 10 and 14 years with a range of different disabilities influenced by risky drinking who are incarcerated, rather than being cared for in the community.	
• There are strong links between the alcohol industry and gambling and they interact to cause social harms.	
• The global alcohol industry and TNCs place their responsibility to shareholders to maintain high level sales above the social and health needs of the Australian community.	
*Natural environment*	
• CUB supports Greenfleet, a leading not-for-profit organisation which restores native biodiverse forests in Australia and New Zealand to capture carbon emissions on behalf of supporters. This helps CUB to calculate and offset vehicle emissions.	
• CUB announced in 2018 that it would adopt 100% renewable energy by 2025	
• In 2018 CUB signed a 12-year Power Purchase Agreement with a German renewable energy developer and service provider for power sourced from a giant solar farm in northern Victoria which provides most of its electricity needs.	
• Solar panels are being installed on the roofs of CUB’s breweries.	
• The global environmental organisation, Greenpeace, used CUB as a case study on positive renewable based energy.	
• CUB is committed to reducing water usage and improving efficiency across its breweries, including a water reclamation facility in Queensland.	
• CUB invests in ‘smart’ agriculture, including new barley varieties to increase quality and to provide long-term commercial opportunities for farmers.	
• CUB has pledged that 100% of its products will be in returnable packaging, or made from mainly recycled content by 2025.	
• In 2019 CUB discontinued six-pack plastic ring packaging which, if discarded, may ensnare marine animals.	
• *However,* alcohol-related littering, including broken glass, still prevails and leads to higher levels of landfill.	
• The brewery process consumes high volumes of water and crop growing usurps arable land that could be used for food production.	
• In 2018 residents in a regional Victorian town lost a court battle to stop CUB’s parent company, Asahi, from engaging a farmer to extract groundwater for bottling; incurring legal fees of Au$90,000.	
*Adverse health impacts*	
• Irreparable damage from FASD results in violence, impacts on relationships, and potential for miscarriage.	
• The transmission of violence and aggression, and a range of other mental health problems continues in a vicious cycle through genetic and early developmental and environmental influences.	
• Alcohol related violence can cause stress and subsequent pre-term labour and low birth weight babies.	
• Aboriginal and Torres Strait Islander populations are more likely to drink alcohol at risky levels but also the most likely to abstain.	
• Mandatory replacement of CUB’s glass bottles with cans in a remote setting with a high Indigenous population has led to a reduction in lacerations, stabbings, and emergency hospital presentations.	
*Economic conditions*	
• CUB’s economic contribution includes directly employing 1700 staff in breweries, distribution centres, and offices. The company supports other jobs in manufacturing, transport, retail, hospitality, tourism and agriculture.	
• Taxation revenue from CUB’s operations provides for health and social investment: a portion of alcohol excise and goods and services taxes of Au$2.4 billion in 2015–2016.	
• *However,* as part of the wider alcohol industry, CUB is also responsible for a portion of the billions of dollars required annually to deal with the health and environmental impacts of alcohol, including the burden on health systems and law enforcement due to vehicle accidents and domestic and other forms of violence, which are externalised to the broader community.	

#### Workforce and working conditions

Carlton and United Breweries currently operates under the 2018–2021 Enterprise Agreement established by the *Fair Work* Act 2009 [73]. CUB’s operational workforce is covered by enterprise agreements negotiated with relevant unions and approved by the Fair Work Commission; with rates of pay exceeding the relevant modern award. The majority of operational staff work a 35 hour week with offers of job sharing, working from home, part-time employment, support for carers, flexible working hours, paid parental leave, and gender inclusivity [61, 74].

However, negative employment practices have also been reported in recent years [75, 76]. In June 2016, CUB sacked 55 highly skilled Melbourne maintenance workers before offering them alternative contractual arrangements as unskilled workers under external labour hire provisions, with greatly reduced wages. This became the subject of a Senate enquiry into corporate avoidance of the *Fair Work Act* [77, 78]. In 2020 the Fair Work Ombudsman investigated CUB’s self-reported unintentional underpayment of $1million affecting 635 hospitality workers over a 10 year period [79].

Workplace health and safety is also a critical employment issue affecting both individual workers, employers, and the broader community [80]. *SafeWork Australia* is Australia’s national work, health and safety policy agency, with regulation governed at state and territory level under 10 separate statutes [81].

SafeWork Queensland noted that the company undertakes continuous improvement in its safety performance by both traditional engineering based approaches and investment in systems and practices [82].

#### Social conditions

The impact of CUB’s operations on social conditions include any effects on local goods and services and local community life. The Brewers Association’s positive framing is that the alcohol industry moderates loneliness and social isolation, especially in rural communities.

However, one in six Australians consumes alcohol at levels placing them at lifetime risk of alcohol related disease or injury [83]. Respondents recounted a range of negative social conditions related to alcohol consumption. One was fear of alcohol-fuelled violence, linked to long trading hours in a regional setting:

*People were literally too afraid to go out of their house at night … What I saw was complete capitulation by the politicians, our elected democratic officials … It was quite clear that they were in the pockets of industry and there were demonstrable harms and an average three or four deaths a year. (Civil society actor #1)*

This respondent also spoke of the negative social conditions, particularly affecting Indigenous communities, which has received national opprobrium [84]:

*We have had the recent case in Darwin, first time the biggest Dan Murphy’s (Woolworths owned liquor outlet) being put between two vulnerable Aboriginal settlements. And we have the battles and the struggles occurring in northwest Western Australia [and Queensland] … We have these nodes of community reaction where the industry is disjointing, profiting, and capitalising from these most vulnerable communities. (Civil society actor #1)*

Subsequently this placement decision was overturned based on findings of a review into the level of community consultation undertaken by Woolworths [85].

Alcohol marketing is a powerful tool, and four companies, including CUB, are responsible for 60% of commercial partnerships. While sporting codes market their sports and events as ‘family-friendly’, they allow direct promotion of products that have negative social and health impacts; especially for young people [86, 87].

#### The natural environment

This part of the CHIA focuses on CUB’s impact on the natural environment including ecological systems, land and water, greenhouse gases and pollution. A positive environmental contribution includes Asahi’s, and thus CUB’s commitment to using green electricity in its Australian and New Zealand operations by 2025. Their aim is to achieve global carbon neutrality by 2050 [88]. Carlton and United Breweries is also a signatory to the Australian Packaging Covenant Organisation (APCO); a voluntary agreement to encourage packaging waste minimisation. It supports Greenfleet, a company which assists CUB to calculate and offset its fleet emissions. The document and media analysis did not identify negative environmental impacts related to CUB’s specific operations. However, respondents noted negative aspects of the broader alcohol industry. These include the high volume of water required to manufacture alcoholic beverages which could instead be used for food production, and the problem of littering. One respondent spoke of the danger of broken beer bottles, or litter that can be employed as weapons; with another graphically recounting the negative local environmental outcomes of risky consumption:

*… all the urine, vomit and blood running down our streets, in [stated city] is not real good. The [named] council spent over $1 million a year on ratepayers’ money, like mine, to clean up after it. (Civil society actor #1) …*

#### Adverse health impacts

This aspect of the CHIA reports on adverse health impacts linked to alcohol consumption. As Livingston and Callinan [18] state, 10% of alcohol consumers account for 50% of consumption; with a respondent (Civil society actor #6) also noting that 80% of alcohol is sold to 20% of consumers. However, common framing by the alcohol industry is that population-wide policies that will affect ‘moderate’ drinkers should not be implemented because harmful consumption is confined to a minority of heavy drinkers, [89].

Alcohol-related violence has major health impacts. Such violence is especially related to venues with late alcohol trading. As Kypri and Livingston [90] explain, alcohol consumption increases the tendency for aggression, leads to misinterpretation of social cues, and impairs problem-solving skills. One participant explained the context of these behaviours:

*We reached the stage where (named city) was a literal bloodbath, we had 20,000 young people come to our CBD every weekend from over 100 km away … They could come here, drink themselves stupid to 5:00 am. There was insufficient police, they were going from one door to another. There was no preventative work and that’s why we had the highest levels of alcohol violence in [the state]. (Civil society actor #1).*

The impacts of Fetal Alcohol Spectrum Disorder, another key issue related to risky drinking behaviours, was highlighted in the research. The Brewers Association has called for a tailored focus on Indigenous communities, rather than adopting population-wide approaches [91]. However, a respondent instead situated FASD as part of a continuum of negative health outcomes that are both community-wide and across the lifespan:

*There are so many particular groups that are affected [by alcohol] that in essence it becomes a whole of community issue … you’ve got the full spectrum of children from unborn through to teenagers. And then alcohol in their early adult years often see increased level of risky drinking. There are the concerns about violence and impacts on young relationship and family relationships … Then you go a bit older you’ve got women of child-bearing age, there are particular impacts again through FASD... Then into adulthood there’s alcohol dependence, there’s alcohol caused cancers and other social and health issues. So really through the whole life span there are risks. (Civil society actor #4)*

#### Economic conditions

This aspect of CHIA refers to CUB’s impact on the national or local economy, production systems, and public revenue. The Brewers Association states that the beer industry alone contributes billions of dollars to government revenue and supports a significant supply chain which supports nearly 100,000 jobs, underpinning $16 billion a year in economic activity. This activity includes beer production, the contribution of the supply chain and the on-license and off-license retailing [92]. Carlton and United Breweries’ website highlights the scope of its own economic contribution by employing approximately 1700 workers in six breweries, distribution centres, and offices around Australia, and its support for thousands of local jobs in transport, retail, hospitality, manufacturing and agriculture.

Although it is not possible to quantify CUB’s overall economic contribution, the Brewers Association notes that its three members (CUB, Lion and Coopers) collectively employ more than 13,500 Australians in brewing, sales, professional services and logistics. Beer taxes raised $3.593 billion in 2018–2019 [92]. However, economic investment is mediated by externalised industry costs estimated to be Au$36 billion annually [10].

A summary of CHIA level C findings on the positive and negative aspects of CUB’s products and operations on daily living conditions across the five domains are included in Table 4.

#### Estimated health impacts: CUB’s share of burden of beer-attributable deaths

As part of the CHIA we conducted an ecological study to estimate alcohol attributable fractions and burdens of death due to congestive heart diseases, diabetes mellitus, stroke, breast cancer, bowel cancer, and injury.

Carlton and United Breweries has had a significant share of the market but in a mixed pattern. The highest beer market share was recorded in 2010, 2020 and 2011/2019; owning 45.7, 44.1 and 43.1% stake respectively. 2016 was the lowest stake with 30.1% [93]. Such a significant share contributed to substantial beer attributable deaths. For example, of the 6.8 deaths per 100,000 population in 2010, 6.2 were alcohol related fatal injuries, and 2.6 deaths were specifically due to beer; of which CUB contributed to 1.2 deaths. In the year 2010, there were 3.3 alcohol related Bowel cancer deaths per 100,000 population and 1.4 deaths were attributed to beer of which CUB’s proportion was 0.6. The cost of treating people with these diseases is mainly externalised to the public purse.

Table 5. describes the alcohol attributable burden and beer attributable burden in Australia from 1990. CUB’s proportion and market share is included from 2010. (See Supplementary Data for full details).

**Table 5:** Alcohol and beer attributable burden of deaths and CUB’s share in Australia

Outcome	Year	Alcohol attributable burden of death	Beer attributable burden of death	CUB’s proportions	CUB’s beer market share
Diabetes mellitus	1990	−20.3	−12.0		
	1991	−23.9	−14.2		
	1992	−21.1	− 12.1		
	1993	−21.3	−11.9		
	1994	−22.8	−12.4		
	1995	− 21.7	−11.8		
	1996	−23.5	−12.7		
	1997	−23.0	−12.2		
	1998	−22.7	−11.7		
	1999	−21.1	−10.8		
	2000	−20.8	−10.6		
	2001	−17.9	−9.0		
	2002	−21.7	−10.6		
	2003	−21.6	− 10.4		
	2004	−24.0	−11.1		
	2005	−21.2	− 9.5		
	2006	−21.5	−9.6		
	2007	−22.7	−9.9		
	2008	−23.1	−10.1		
	2009	− 22.4	−9.7		
	2010	−20.3	−8.6	−3.9	45.7
	2011	−16.0	−6.6	−2.8	43.1
	2012	−17.8	−7.3	−2.5	34.7
	2013	−23.7	−9.6	−3.6	37.6
	2014	−20.1	−8.1	−2.9	35.7
	2015	−21.0	−8.2	− 3.1	38.6
	2016	−24.5	−9.7	−2.9	30.1
Congestive heart disease	1990	−48.8	−28.9		
	1991	−49.6	−29.5		
	1992	−46.7	−26.8		
	1993	−42.1	−23.6		
	1994	−42.8	−23.2		
	1995	−40.1	−21.9		
	1996	−39.0	−21.1		
	1997	−37.5	−19.9		
	1998	−36.1	−18.6		
	1999	−33.0	−16.8		
	2000	−30.5	−15.5		
	2001	−26.9	−13.5		
	2002	−28.1	−13.7		
	2003	−26.7	−12.9		
	2004	−26.1	−12.0		
	2005	−23.3	−10.4		
	2006	−22.1	−9.9		
	2007	−21.7	−9.5		
	2008	−21.3	−9.3		
	2009	−19.6	−8.4		
	2010	−18.1	−7.6	−3.5	45.7
	2011	−14.9	−6.1	−2.6	43.1
	2012	−14.5	−5.9	−2.1	34.7
	2013	−16.1	−6.5	−2.5	37.6
	2014	−14.9	−6.0	−2.2	35.7
	2015	−14.3	−5.6	−2.1	38.6
	2016	−14.5	−5.7	−1.7	30.1
Stroke	1990	2.7	1.6		
	1991	2.7	1.6		
	1992	2.5	1.4		
	1993	2.4	1.4		
	1994	2.5	1.4		
	1995	2.4	1.3		
	1996	2.3	1.3		
	1997	1.9	1.0		
	1998	1.8	1.0		
	1999	1.7	0.9		
	2000	1.7	0.8		
	2001	1.4	0.7		
	2002	1.5	0.7		
	2003	1.5	0.7		
	2004	1.5	0.7		
	2005	1.3	0.6		
	2006	1.3	0.6		
	2007	1.3	0.6		
	2008	1.2	0.5		
	2009	1.1	0.5		
	2010	1.1	0.5	0.2	
	2011	1.0	0.4	0.2	
	2012	1.0	0.4	0.1	
	2013	1.0	0.4	0.2	
	2014	1.0	0.4	0.1	
	2015	0.9	0.4	0.1	
	2016	1.0	0.4	0.1	
Breast cancer	1990	3.3	1.9		
	1991	3.2	1.9		
	1992	3.1	1.8		
	1993	3.2	1.8		
	1994	3.2	1.7		
	1995	3.1	1.7		
	1996	3.0	1.6		
	1997	2.9	1.5		
	1998	2.9	1.5		
	1999	2.6	1.3		
	2000	2.6	1.3		
	2001	2.4	1.2		
	2002	2.6	1.3		
	2003	2.6	1.2		
	2004	2.6	1.2		
	2005	2.5	1.1		
	2006	2.3	1.0		
	2007	2.4	1.1		
	2008	2.1	0.9		
	2009	2.3	1.0		
	2010	2.3	1.0	0.4	45.7
	2011	2.1	0.9	0.4	43.1
	2012	2.0	0.8	0.3	34.7
	2013	2.1	0.9	0.3	37.6
	2014	2.1	0.9	0.3	35.7
	2015	2.1	0.8	0.3	38.6
	2016	2.1	0.8	0.2	30.1
Bowel cancer	1990	4.5	2.6		
	1991	4.7	2.8		
	1992	4.4	2.5		
	1993	4.3	2.4		
	1994	4.5	2.4		
	1995	4.2	2.3		
	1996	4.3	2.3		
	1997	4.2	2.2		
	1998	4.2	2.1		
	1999	4.0	2.0		
	2000	3.9	2.0		
	2001	3.6	1.8		
	2002	3.7	1.8		
	2003	3.7	1.8		
	2004	3.6	1.7		
	2005	3.5	1.6		
	2006	3.4	1.5		
	2007	3.4	1.5		
	2008	3.3	1.4		
	2009	3.3	1.4		
	2010	3.2	1.3	0.6	45.7
	2011	2.7	1.1	0.5	43.1
	2012	2.8	1.2	0.4	34.7
	2013	3.1	1.3	0.5	37.6
	2014	2.9	1.2	0.4	35.7
	2015	2.9	1.1	0.4	38.6
	2016	3.0	1.2	0.4	30.1
Injury	1990	14.1	8.4		
	1991	12.4	7.4		
	1992	11.5	6.6		
	1993	10.7	6.0		
	1994	10.6	5.8		
	1995	11.0	6.0		
	1996	10.3	5.6		
	1997	9.6	5.1		
	1998	9.3	4.8		
	1999	9.2	4.7		
	2000	9.2	4.7		
	2001	8.9	4.5		
	2002	8.6	4.2		
	2003	7.9	3.8		
	2004	7.3	3.4		
	2005	7.1	3.2		
	2006	7.6	3.4		
	2007	7.1	3.1		
	2008	6.5	2.8		
	2009	6.6	2.9		
	2010	6.2	2.6	1.2	45.7
	2011	5.6	2.3	1.0	43.1
	2012	5.5	2.2	0.8	34.7
	2013	5.2	2.1	0.8	37.6
	2014	5.0	2.0	0.7	35.7
	2015	5.0	1.9	0.7	38.6
	2016	5.1	2.0	0.6	30.1

## Discussion

Carlton and United Breweries’ operations are conducted under a neoliberal regulatory environment reflecting the changing face of twenty-first century capitalism that promotes private interests ahead of population health [29, 94]. Freudenberg [95] describes the evolution of the ‘corporate-consumption complex’ which can be extrapolated to our research. This is the web of organisations such as TNCs that produce alcoholic products; retail conglomerates or alcohol megastores; the trade associations that represent alcohol TNCs politically, such as the ABA and AHA; and lobbying groups such as the Brewers Association of Australia, all highlighted in our research. Our CHIA thus demonstrated the ways in which CUB plays a role in this complex web. The research also highlighted how elected officials, another part of this complex, may be subject to regulatory capture by the alcohol industry [3].

Our CHIA identified actual and potential positive health impacts. These include CUB’s investment in sustainable operations, employment opportunities, work health and safety standards, and a range of beneficial employment conditions. CUB’s products can provide opportunities for enjoyable and relatively affordable social interactions; especially in regional communities where the local hotel remains an important community meeting place. CUB’s range of philanthropic initiatives which benefit communities is likely to be positive for health.

However, actual and potential negative health impacts reflecting the addictive nature of alcohol were also reported [83]. Carlton and United Breweries’ Australian beer market share, approximating 45%, highlights the proportional responsibility that CUB must take for the attributable fractions and health burdens measured. Carlton and United Breweries is also proportionally responsible for externalised health costs related to a range of diseases, different forms of violence, FASD, and burdens on policing operations and child protection services.

The CHIA also noted the shortcomings of self-regulation which does not properly protect children and young people from exposure to powerful marketing; especially through CUB’s major sponsorship of popular sporting codes. Voluntary codes for alcohol TNC operations have little public accountability; relying instead on reactive reputation management.

Our research informs existing debates about the alcohol industry, as examining both positive and negative practices of one particular TNC can help to identify the extent to which it, and potentially other alcohol TNCs, may impact health and health equity. In turn, this may assist governments, civil society actors and TNCs to take action to promote positive and reduce negative aspects of industry operations. This research on an alcohol TNC adds to our prior research on a fast food TNC in Australia, and an extractives TNC in Australia and Southern Africa to help draw similarities and differences between corporate strategies across different industry sectors.

Public health researchers must focus on the health impacts of corporate products, but also interrogate their broader practices and their relationship with the broader global political economy.

Another changing dynamic in alcohol and other industries, is the power and proliferation of large [liquor] outlets, as highlighted in our study. Overall, CUB’s core business is selling alcohol and it is responsible for approximately 45% of alcohol sold in Australia. While people with higher SES may consume similar or greater amounts of alcohol compared with lower SES, the latter appears to bear a disproportionate burden of negative outcomes. These are further complicated by other moderating factors including race, ethnicity, gender, and homelessness [96].

Carlton and United Breweries’ influence on alcohol regulation (including advertising, licensing, and health warnings) has a probable major negative impact on health and wellbeing with disproportionate negative impact on young people. It also has a probable minor or moderate positive impact on employment opportunities. Carlton and United Breweries’ probable minor positive impact on the natural environment through sustainable practices is possibly outweighed by the overall negative impact on the environment through production and distribution of alcohol. There is also a similar pattern in regards to community benefits (e.g. philanthropic contributions and social benefits), with the potential positive benefits being significantly outweighed by the harms caused by alcohol consumption and externalised costs of addressing these harms.

Carlton and United Breweries’ business practices, in particular their probable moderate influence on alcohol regulation, has a probable moderate (significant) negative impact on Australia’s capacity to enact evidence-based actions that can mitigate alcohol related harm. In particular these are alcohol pricing; limits on alcohol advertising and sponsorship; licensing including outlet density and opening hours; and alcohol warning labels.

### Research strengths and limitations

The strength of this research was applying the CHIA framework to a third industry sector with a complex history of ownership changes following our research on a fast-food and an extractives corporation. This allowed for consolidating cross-sector information on similarities and differences relating to corporate operations and health impacts. CUB’s successive ownership changes provided additional insights into the phenomenon of asset churning within twenty-first century capitalism and how this complexity imposes constraints on conducting CHIAs.

The refusal of both CUB and other industry representatives to engage in the research is ostensibly a limitation. However, this is consistent with our prior research [36, 37] and an earlier study conducted by others on Walmart [97] which shows that assessment of a TNC’s health impacts does not depend on participation by TNC or industry-representative staff. Our study also demonstrated the challenges of conducting a comprehensive CHIA. While we found substantial evidence to describe some of the impacts of CUB, other information that could have informed this analysis was proprietary or not available for review by independent researchers. In the future, public health researchers, public health agencies and other government officials will need to find additional ways to conduct comprehensive assessments of the health impact of specific corporations.

The ecological study which estimated attributable alcohol risk, including CUB’s share, used mortality data for the analyses; improvements in which occurred over time as a function of earlier disease identification, improved treatment modalities and therefore increased survival over time. For this reason, the estimates of mortality burden were highly likely to have been underestimated in more recent years. Further, the analyses were restricted to published estimates of relative risk, which were derived from studies known to be subject to significant methodological weaknesses, specifically in relation to cardiovascular disease mortality [45]. These methodological weaknesses, combined with differential reporting of estimates of uncertainty across studies, are reflected in our omission of estimates of uncertainty, and all ecological outcomes should be interpreted cautiously.

Evidence suggests that financial institutions, banks and private equity funds play a critical role in shaping businesses and business practices; especially over the last two decades. Our research did not consider the extent to which these institutions exert power in respect of changing ownership of TNCs, and how this may affect the managers of companies such as CUB facing acquisition by successive parent bodies. We did not find sufficient evidence in our documents or interviews to make an assessment of CUB’s health equity impacts which highlights another important area for future research. Some of the negative consequences of financialization are exacerbating inequalities, greater insecurity, and the erosion of trust [98] which suggest directions for future research with a strong equity focus.

## Conclusion

Studying a TNC in a rapidly changing global financialized capitalist economy, in a world in which globalisation is increasingly being managed by TNCs in many facets of life, poses methodological and conceptual challenges. Our study of an Australian alcohol company operating under the changing face of twenty-first century capitalism highlights the opportunity for future research on TNCs. The alcohol industry offers some benefits, but these are won at the expense of significant harms. Australia’s alcohol consumption is relatively high, and CUB is a leading Australian alcohol company. It therefore acts as a commercial determinant of health (CDoH), and further regulation on products, advertising, sponsorship, and political engagement would be beneficial from a public health perspective.

## Supplementary Information

**Additional file 1.**

## Data Availability

Full information on the epidemiological analysis of Estimated Health Impacts is available in the Supplementary Data. The qualitative datasets used or analysed during the current study are available from the corresponding author on reasonable request.

## Abbreviations

ABA: Australian Beverages Association
ABAC: Alcohol Beverages Advertising Code
ABS: Australian Bureau of Statistics
AHA: Australian Hotels Association
APCO: Australian Packaging Convention Organisation
CDOH: Commercial determinants of health
CHIA: Corporate health impact assessment
CUB: Carlton and United Breweries
FASD: Fetal Alcohol Spectrum Disorder
FSANZ: Food Standards Australia and New Zealand
GHG: Greenhouse gas
HIA: Health impact assessment
PR_d_: Prevalence of drinking
RR: Relative risk
SAPRO: Social Aspects and Public Relations Organisation
TNC: Transnational corporation
