# Supplementary material for: Assessing the health impacts of transnational corporations: a case study of Carlton and United Breweries in Australia
Source: Global Health. 2022 Sep 9;18:80. doi: 10.1186/s12992-022-00870-0 (PMC9462641; doi:10.1186/s12992-022-00870-0)
Supplement: Supplementary file 1 — Additional file 1. [file 12992_2022_870_MOESM1_ESM.docx]

**METHODS**

**Study design**

This study follows a mixed methods approach including an ecological study, document review and qualitative interviews. The ecological study involved estimating alcohol attributable fractions (PAF) and burden of deaths due to congested heart diseases (CHD), diabetes mellitus (DM), stroke, breast cancer, bowel cancer and injury. Moreover, beer attributable fractions and deaths and CUB’s share for the aforementioned chronic illnesses and injury were also estimated.

**Data sources**

The indicators used in the ecological study included: age standardised mortality, prevalence of drinking (PR_d_), alcohol (and beer) consumption in volume of pure alcohol, beer per capita consumption of pure alcohol, CUB’s beer share, and relative risk (RR) of alcohol consumption for the abovementioned chronic illnesses and injury.

Our analyses included age standardised mortality per 100,000 population from the Australian Institute of Health and Welfare (AIHW).^1^ The sources of PR_d_ for 1990, 2000 and 2005 for Australia were utilised from the 2016 systematic analysis for the Global Burden of Disease Study;^2^ for 1998, 2001, 2004, 2007, 2010, 2013 and 2016 from Australia’s National Drug Strategy Household Survey; and for 2008, 2012 and 2014 from Australian Bureau of Statistics (ABS).^3^ We applied mean imputation for the years 1991-1997, 1999, 2003, 2006, 2009, 2011 and 2015. Data for PR_d_ are presented in Supplementary Table 1. The alcohol (and beer) consumption in volume of pure alcohol in ‘000s of litres and per capita consumption of pure alcohol were obtained from ABS.^3^ Data for CUB’s market beer share were obtained from *IBISWorld* data^4^ (only available for 2010 onwards, Table 1).

We searched meta-analytic studies from PubMed and relevant bibliographies to find the RR using the following search concepts: (i) alcohol consumption, (ii) CHD, DM, stroke, breast cancer, bowel cancer and injury, and (iii) systematic review/meta-analysis. Given the RR are different for different levels of alcohol consumption, we considered the RR for Australia’s average consumption level as the cut-off. The ABS reported that the average consumption for Australias is 2.72 standard drinks, or 27.2 grams of alcohol, per day.^5^ Accordingly, the RRs for 27.2 gram per day consumption of alcohol for CHD,^6^ DM,^7^ stroke,^8^ breast cancer,^9^ bowel cancer^10^ and injury^11^ were 0.8, 0.36, 1.04, 1.14, 1.2, and 13 respectively.

**Attributable burden due to alcohol use**

We calculated PAF using the following formula.

$PAF=\frac{Prevalence of drinking \boldsymbol{X} (RR-1)}{Prevalence of drinking \boldsymbol{X} \left( RR-1 \right)+1}$ …………………………………………Formula (1)

PAF of 1 (100%) is assumed as fully attributable. Negative PAF values indicate that alcohol is a protective factor. Following the PAF calculation, PAFs were multiplied by outcome-specific age standardised estimates of deaths to calculate the total attributable burden for each outcome in each specific period. To calculate the beer attributable burden for each outcome, we multiplied the total alcohol attributable outcome specific burden by the proportion of volume of alcohol in beer per capita. To quantify CUB’s contribution, we multiplied the beer attributable burden by CUB’s beer share proportion.

**RESULTS**

**Age standardised mortality rates per 100,000 population**

The age standardized mortality in Australia is described in Figure 1. The overall standardized mortality rates were declining over time. CHD contributes to significant death followed by stroke; however, injury and DM contributed the least.

### Figure 1. Age standardised mortality rate per 100,000 population in Australia, 1990-2016

### Patterns in the burden of alcohol-attributable deaths

### The PAFs for CHD and DM were found to be negative, but positive for stroke, breast cancer, bowel cancer and injury (Supplementary Table 1). Figure 2 describes the trend of alcohol attributable deaths. More deaths could have been saved from injury and bowel cancer than stroke by avoiding alcohol consumption. For example, alcohol contributed to 14.1 and 5.1 fatal injuries in 1990 and 2016 respectively, but 2.7 and 1.0 stroke related deaths in those periods. Interestingly, the data showed an inverse relationship between alcohol and mortality due to CHD and DM. Overall, alcohol attributable deaths declined from 1990 to 2016.

###

### Figure 2. Patterns of alcohol attributable deaths rate per 100,000 population in Australia, 1990-2016

### Patterns in burden of beer-attributable deaths

### Figure 3 describes the trend beer attributable deaths. More deaths could have been saved from injury and bowel cancer than stroke by avoiding beer consumption. Moreover, the contribution of beer was substantial. For example, beer contributed to 8.4 of 14.1 alcohol related fatal injuries in 1990; and 2 of 5.1 alcohol related fatal injuries in 2016. Similar to overall alcohol, the data showed an inverse relationship between beer and mortality due to CHD and DM.

### Figure 3. Patterns of beer alcohol attributable deaths rate per 100,000 population in Australia, 1990-2016

**CUB’s share to burden of beer-attributable deaths**

The *IBISWorld* shows that CUB has had significant share but in a mixed pattern. The highest beer market share was recorded in 2010, 2020 and 2011/2019 owning 45.7%, 44.1% and 43.1% stake respectively but 2016 was the lowest stake with 30.1%. Such significant share contributed to substantial beer attributable deaths. For example, Of the 6.8 deaths per 100,000 population in 2010, 6.2 were alcohol related fatal injuries, and 2.6 deaths were specifically due to beer of which CUB contributed to 1.2 deaths. Table 1 describes the alcohol attributable burden, beer attributable burden, CUB’s proportion, and CUB’s beer market share.

### Table 1. Alcohol and beer attributable burden of deaths and CUB’s share in Australia

| Outcome | Year | Age standardized deaths | Alcohol attributable burden of death | Beer attributable burden of death | CUB’s proportions | CUB’s beer market share |
| --- | --- | --- | --- | --- | --- | --- |
| Diabetes mellitus | 1990 | 15.7 | -20.3 | -12.0 |  |  |
|  | 1991 | 16.0 | -23.9 | -14.2 |  |  |
|  | 1992 | 16.3 | -21.1 | -12.1 |  |  |
|  | 1993 | 16.9 | -21.3 | -11.9 |  |  |
|  | 1994 | 17.6 | -22.8 | -12.4 |  |  |
|  | 1995 | 16.9 | -21.7 | -11.8 |  |  |
|  | 1996 | 18.2 | -23.5 | -12.7 |  |  |
|  | 1997 | 17.8 | -23.0 | -12.2 |  |  |
|  | 1998 | 16.4 | -22.7 | -11.7 |  |  |
|  | 1999 | 16.3 | -21.1 | -10.8 |  |  |
|  | 2000 | 16.1 | -20.8 | -10.6 |  |  |
|  | 2001 | 16.0 | -17.9 | -9.0 |  |  |
|  | 2002 | 16.8 | -21.7 | -10.6 |  |  |
|  | 2003 | 16.7 | -21.6 | -10.4 |  |  |
|  | 2004 | 17.3 | -24.0 | -11.1 |  |  |
|  | 2005 | 16.4 | -21.2 | -9.5 |  |  |
|  | 2006 | 16.6 | -21.5 | -9.6 |  |  |
|  | 2007 | 16.7 | -22.7 | -9.9 |  |  |
|  | 2008 | 17.8 | -23.1 | -10.1 |  |  |
|  | 2009 | 17.3 | -22.4 | -9.7 |  |  |
|  | 2010 | 15.8 | -20.3 | -8.6 | -1.4 | 45.7 |
|  | 2011 | 16.3 | -16.0 | -6.6 | -1.1 | 43.1 |
|  | 2012 | 16.0 | -17.8 | -7.3 | -1.0 | 34.7 |
|  | 2013 | 15.9 | -23.7 | -9.6 | -1.2 | 37.6 |
|  | 2014 | 15.5 | -20.1 | -8.1 | -1.0 | 35.7 |
|  | 2015 | 16.3 | -21.0 | -8.2 | -1.1 | 38.6 |
|  | 2016 | 16.2 | -24.5 | -9.7 | -1.0 | 30.1 |
| Congestive heart disease | 1990 | 228.1 | -48.8 | -28.9 |  |  |
|  | 1991 | 215.6 | -49.6 | -29.5 |  |  |
|  | 1992 | 218.2 | -46.7 | -26.8 |  |  |
|  | 1993 | 199.9 | -42.1 | -23.6 |  |  |
|  | 1994 | 200.3 | -42.8 | -23.2 |  |  |
|  | 1995 | 188.1 | -40.1 | -21.9 |  |  |
|  | 1996 | 182.4 | -39.0 | -21.1 |  |  |
|  | 1997 | 175.4 | -37.5 | -19.9 |  |  |
|  | 1998 | 163.3 | -36.1 | -18.6 |  |  |
|  | 1999 | 154.1 | -33.0 | -16.8 |  |  |
|  | 2000 | 142.8 | -30.5 | -15.5 |  |  |
|  | 2001 | 136.0 | -26.9 | -13.5 |  |  |
|  | 2002 | 131.2 | -28.1 | -13.7 |  |  |
|  | 2003 | 124.9 | -26.7 | -12.9 |  |  |
|  | 2004 | 117.7 | -26.1 | -12.0 |  |  |
|  | 2005 | 109.0 | -23.3 | -10.4 |  |  |
|  | 2006 | 103.5 | -22.1 | -9.9 |  |  |
|  | 2007 | 99.0 | -21.7 | -9.5 |  |  |
|  | 2008 | 99.4 | -21.3 | -9.3 |  |  |
|  | 2009 | 91.4 | -19.6 | -8.4 |  |  |
|  | 2010 | 84.8 | -18.1 | -7.6 | -3.5 | 45.7 |
|  | 2011 | 81.2 | -14.9 | -6.1 | -2.6 | 43.1 |
|  | 2012 | 73.5 | -14.5 | -5.9 | -2.0 | 34.7 |
|  | 2013 | 70.2 | -16.1 | -6.5 | -2.4 | 37.6 |
|  | 2014 | 69.7 | -14.9 | -6.0 | -2.1 | 35.7 |
|  | 2015 | 66.9 | -14.3 | -5.6 | -2.2 | 38.6 |
|  | 2016 | 62.4 | -14.5 | -5.7 | -1.7 | 30.1 |
| Stroke | 1990 | 79.9 | 2.7 | 1.6 |  |  |
|  | 1991 | 76.1 | 2.7 | 1.6 |  |  |
|  | 1992 | 74.1 | 2.5 | 1.4 |  |  |
|  | 1993 | 72.8 | 2.4 | 1.4 |  |  |
|  | 1994 | 73.4 | 2.5 | 1.4 |  |  |
|  | 1995 | 70.0 | 2.4 | 1.3 |  |  |
|  | 1996 | 68.9 | 2.3 | 1.3 |  |  |
|  | 1997 | 54.9 | 1.9 | 1.0 |  |  |
|  | 1998 | 52.8 | 1.8 | 1.0 |  |  |
|  | 1999 | 51.3 | 1.7 | 0.9 |  |  |
|  | 2000 | 48.6 | 1.7 | 0.8 |  |  |
|  | 2001 | 45.2 | 1.4 | 0.7 |  |  |
|  | 2002 | 45.1 | 1.5 | 0.7 |  |  |
|  | 2003 | 44.1 | 1.5 | 0.7 |  |  |
|  | 2004 | 41.8 | 1.5 | 0.7 |  |  |
|  | 2005 | 37.8 | 1.3 | 0.6 |  |  |
|  | 2006 | 37.9 | 1.3 | 0.6 |  |  |
|  | 2007 | 37.0 | 1.3 | 0.6 |  |  |
|  | 2008 | 36.5 | 1.2 | 0.5 |  |  |
|  | 2009 | 33.3 | 1.1 | 0.5 |  |  |
|  | 2010 | 32.2 | 1.1 | 0.5 | 0.2 | 45.7 |
|  | 2011 | 33.1 | 1.0 | 0.4 | 0.2 | 43.1 |
|  | 2012 | 30.4 | 1.0 | 0.4 | 0.1 | 34.7 |
|  | 2013 | 28.5 | 1.0 | 0.4 | 0.2 | 37.6 |
|  | 2014 | 28.3 | 1.0 | 0.4 | 0.1 | 35.7 |
|  | 2015 | 27.9 | 0.9 | 0.4 | 0.2 | 38.6 |
|  | 2016 | 26.9 | 1.0 | 0.4 | 0.1 | 30.1 |
| Breast cancer | 1990 | 31.0 | 3.3 | 1.9 |  |  |
|  | 1991 | 31.1 | 3.2 | 1.9 |  |  |
|  | 1992 | 29.5 | 3.1 | 1.8 |  |  |
|  | 1993 | 31.2 | 3.2 | 1.8 |  |  |
|  | 1994 | 30.7 | 3.2 | 1.7 |  |  |
|  | 1995 | 29.6 | 3.1 | 1.7 |  |  |
|  | 1996 | 28.8 | 3.0 | 1.6 |  |  |
|  | 1997 | 28.0 | 2.9 | 1.5 |  |  |
|  | 1998 | 26.7 | 2.9 | 1.5 |  |  |
|  | 1999 | 25.5 | 2.6 | 1.3 |  |  |
|  | 2000 | 24.8 | 2.6 | 1.3 |  |  |
|  | 2001 | 24.9 | 2.4 | 1.2 |  |  |
|  | 2002 | 25.4 | 2.6 | 1.3 |  |  |
|  | 2003 | 24.9 | 2.6 | 1.2 |  |  |
|  | 2004 | 23.7 | 2.6 | 1.2 |  |  |
|  | 2005 | 23.9 | 2.5 | 1.1 |  |  |
|  | 2006 | 22.3 | 2.3 | 1.0 |  |  |
|  | 2007 | 22.3 | 2.4 | 1.1 |  |  |
|  | 2008 | 22.6 | 2.1 | 0.9 |  |  |
|  | 2009 | 22.1 | 2.3 | 1.0 |  |  |
|  | 2010 | 21.8 | 2.3 | 1.0 | 0.5 | 45.7 |
|  | 2011 | 21.9 | 2.1 | 0.9 | 0.4 | 43.1 |
|  | 2012 | 20.6 | 2.0 | 0.8 | 0.3 | 34.7 |
|  | 2013 | 20.5 | 2.1 | 0.9 | 0.3 | 37.6 |
|  | 2014 | 19.6 | 2.1 | 0.9 | 0.3 | 35.7 |
|  | 2015 | 20.1 | 2.1 | 0.8 | 0.3 | 38.6 |
|  | 2016 | 19.9 | 2.1 | 0.8 | 0.2 | 30.1 |
| Bowel cancer | 1990 | 29.7 | 4.6 | 2.7 |  |  |
|  | 1991 | 29.5 | 4.8 | 2.9 |  |  |
|  | 1992 | 29.4 | 4.6 | 2.6 |  |  |
|  | 1993 | 29.3 | 4.5 | 2.5 |  |  |
|  | 1994 | 29.9 | 4.7 | 2.5 |  |  |
|  | 1995 | 28.4 | 4.4 | 2.4 |  |  |
|  | 1996 | 28.5 | 4.4 | 2.4 |  |  |
|  | 1997 | 28.2 | 4.4 | 2.3 |  |  |
|  | 1998 | 27.2 | 4.3 | 2.2 |  |  |
|  | 1999 | 26.6 | 4.2 | 2.1 |  |  |
|  | 2000 | 26.3 | 4.1 | 2.1 |  |  |
|  | 2001 | 25.7 | 3.8 | 1.9 |  |  |
|  | 2002 | 24.7 | 3.9 | 1.9 |  |  |
|  | 2003 | 24.7 | 3.9 | 1.9 |  |  |
|  | 2004 | 23.4 | 3.8 | 1.7 |  |  |
|  | 2005 | 23.4 | 3.6 | 1.6 |  |  |
|  | 2006 | 22.6 | 3.5 | 1.6 |  |  |
|  | 2007 | 22.1 | 3.5 | 1.5 |  |  |
|  | 2008 | 22.2 | 3.5 | 1.5 |  |  |
|  | 2009 | 22.0 | 3.4 | 1.5 |  |  |
|  | 2010 | 21.1 | 3.3 | 1.4 | 0.6 | 45.7 |
|  | 2011 | 20.2 | 2.8 | 1.2 | 0.5 | 43.1 |
|  | 2012 | 20.0 | 2.9 | 1.2 | 0.4 | 34.7 |
|  | 2013 | 19.8 | 3.2 | 1.3 | 0.5 | 37.6 |
|  | 2014 | 19.1 | 3.0 | 1.2 | 0.4 | 35.7 |
|  | 2015 | 19.2 | 3.0 | 1.2 | 0.5 | 38.6 |
|  | 2016 | 18.8 | 3.1 | 1.2 | 0.4 | 30.1 |
| Injury | 1990 | 15.5 | 14.1 | 8.4 |  |  |
|  | 1991 | 13.5 | 12.4 | 7.4 |  |  |
|  | 1992 | 12.6 | 11.5 | 6.6 |  |  |
|  | 1993 | 11.7 | 10.7 | 6.0 |  |  |
|  | 1994 | 11.7 | 10.6 | 5.8 |  |  |
|  | 1995 | 12.0 | 11.0 | 6.0 |  |  |
|  | 1996 | 11.3 | 10.3 | 5.6 |  |  |
|  | 1997 | 10.5 | 9.6 | 5.1 |  |  |
|  | 1998 | 10.1 | 9.3 | 4.8 |  |  |
|  | 1999 | 10.1 | 9.2 | 4.7 |  |  |
|  | 2000 | 10.1 | 9.2 | 4.7 |  |  |
|  | 2001 | 9.8 | 8.9 | 4.5 |  |  |
|  | 2002 | 9.4 | 8.6 | 4.2 |  |  |
|  | 2003 | 8.6 | 7.9 | 3.8 |  |  |
|  | 2004 | 8.0 | 7.3 | 3.4 |  |  |
|  | 2005 | 7.8 | 7.1 | 3.2 |  |  |
|  | 2006 | 8.3 | 7.6 | 3.4 |  |  |
|  | 2007 | 7.8 | 7.1 | 3.1 |  |  |
|  | 2008 | 7.1 | 6.5 | 2.8 |  |  |
|  | 2009 | 7.2 | 6.6 | 2.9 |  |  |
|  | 2010 | 6.8 | 6.2 | 2.6 | 1.2 | 45.7 |
|  | 2011 | 6.2 | 5.6 | 2.3 | 1.0 | 43.1 |
|  | 2012 | 6.0 | 5.5 | 2.2 | 0.8 | 34.7 |
|  | 2013 | 5.7 | 5.2 | 2.1 | 0.8 | 37.6 |
|  | 2014 | 5.5 | 5.0 | 2.0 | 0.7 | 35.7 |
|  | 2015 | 5.4 | 5.0 | 1.9 | 0.7 | 38.6 |
|  | 2016 | 5.6 | 5.1 | 2.0 | 0.6 | 30.1 |

### Limitations

### We used mortality data for our analyses, improvements in which occurred over time as a function of earlier disease identification, improved treatment modalities and therefore increased survival over time. For this reason, the estimates of mortality burden were highly likely to have been underestimated in more recent years. Further, the analyses were restricted to published estimates of relative risk, which were derived from studies known to be subject to significant methodological weaknesses, specifically in relation to cardiovascular disease mortality.^6^ Finally, as with all ecological studies, the approach is subject to the ‘ecological fallacy’ in which findings in regard to populations does not necessarily apply to individuals, who will consume alcohol at levels that vary from the population average and whose health response will differ according to their physiology, socioeconomic status and ethnicity.

### Supplementary table 1

| Outcome | Year | PR_d_ | Mortality | RR | PAF | PAR _alcohol_ | PAR _beer_ |
| --- | --- | --- | --- | --- | --- | --- | --- |
| Diabetes mellitus | 1990 | 88.1 | 15.7 | 0.36 | -1.293 | -20.3 | -12.0 |
|  | 1991 | 93.5 | 16.0 | 0.36 | -1.490 | -23.9 | -14.2 |
|  | 1992 | 88.1 | 16.3 | 0.36 | -1.293 | -21.1 | -12.1 |
|  | 1993 | 87 | 16.9 | 0.36 | -1.256 | -21.3 | -11.9 |
|  | 1994 | 88.1 | 17.6 | 0.36 | -1.293 | -22.8 | -12.4 |
|  | 1995 | 87.8 | 16.9 | 0.36 | -1.283 | -21.7 | -11.8 |
|  | 1996 | 88.1 | 18.2 | 0.36 | -1.293 | -23.5 | -12.7 |
|  | 1997 | 88.1 | 17.8 | 0.36 | -1.293 | -23.0 | -12.2 |
|  | 1998 | 90.6 | 16.4 | 0.36 | -1.380 | -22.7 | -11.7 |
|  | 1999 | 88.1 | 16.3 | 0.36 | -1.293 | -21.1 | -10.8 |
|  | 2000 | 88.1 | 16.1 | 0.36 | -1.293 | -20.8 | -10.6 |
|  | 2001 | 82.5 | 16.0 | 0.36 | -1.119 | -17.9 | -9.0 |
|  | 2002 | 88.1 | 16.8 | 0.36 | -1.293 | -21.7 | -10.6 |
|  | 2003 | 88.1 | 16.7 | 0.36 | -1.293 | -21.6 | -10.4 |
|  | 2004 | 90.7 | 17.3 | 0.36 | -1.384 | -24.0 | -11.1 |
|  | 2005 | 88.1 | 16.4 | 0.36 | -1.293 | -21.2 | -9.5 |
|  | 2006 | 88.1 | 16.6 | 0.36 | -1.293 | -21.5 | -9.6 |
|  | 2007 | 89.9 | 16.7 | 0.36 | -1.355 | -22.7 | -9.9 |
|  | 2008 | 88.2 | 17.8 | 0.36 | -1.296 | -23.1 | -10.1 |
|  | 2009 | 88.1 | 17.3 | 0.36 | -1.293 | -22.4 | -9.7 |
|  | 2010 | 87.9 | 15.8 | 0.36 | -1.286 | -20.3 | -8.6 |
|  | 2011 | 77.4 | 16.3 | 0.36 | -0.982 | -16.0 | -6.6 |
|  | 2012 | 82.4 | 16.0 | 0.36 | -1.116 | -17.8 | -7.3 |
|  | 2013 | 93.5 | 15.9 | 0.36 | -1.490 | -23.7 | -9.6 |
|  | 2014 | 88.1 | 15.5 | 0.36 | -1.293 | -20.1 | -8.1 |
|  | 2015 | 88.1 | 16.3 | 0.36 | -1.293 | -21.0 | -8.2 |
|  | 2016 | 94.1 | 16.2 | 0.36 | -1.514 | -24.5 | -9.7 |
| Congestive heart failure | 1990 | 88.1 | 228.1 | 0.8 | -0.214 | -48.8 | -28.9 |
|  | 1991 | 93.5 | 215.6 | 0.8 | -0.230 | -49.6 | -29.5 |
|  | 1992 | 88.1 | 218.2 | 0.8 | -0.214 | -46.7 | -26.8 |
|  | 1993 | 87 | 199.9 | 0.8 | -0.211 | -42.1 | -23.6 |
|  | 1994 | 88.1 | 200.3 | 0.8 | -0.214 | -42.8 | -23.2 |
|  | 1995 | 87.8 | 188.1 | 0.8 | -0.213 | -40.1 | -21.9 |
|  | 1996 | 88.1 | 182.4 | 0.8 | -0.214 | -39.0 | -21.1 |
|  | 1997 | 88.1 | 175.4 | 0.8 | -0.214 | -37.5 | -19.9 |
|  | 1998 | 90.6 | 163.3 | 0.8 | -0.221 | -36.1 | -18.6 |
|  | 1999 | 88.1 | 154.1 | 0.8 | -0.214 | -33.0 | -16.8 |
|  | 2000 | 88.1 | 142.8 | 0.8 | -0.214 | -30.5 | -15.5 |
|  | 2001 | 82.5 | 136.0 | 0.8 | -0.198 | -26.9 | -13.5 |
|  | 2002 | 88.1 | 131.2 | 0.8 | -0.214 | -28.1 | -13.7 |
|  | 2003 | 88.1 | 124.9 | 0.8 | -0.214 | -26.7 | -12.9 |
|  | 2004 | 90.7 | 117.7 | 0.8 | -0.222 | -26.1 | -12.0 |
|  | 2005 | 88.1 | 109.0 | 0.8 | -0.214 | -23.3 | -10.4 |
|  | 2006 | 88.1 | 103.5 | 0.8 | -0.214 | -22.1 | -9.9 |
|  | 2007 | 89.9 | 99.0 | 0.8 | -0.219 | -21.7 | -9.5 |
|  | 2008 | 88.2 | 99.4 | 0.8 | -0.214 | -21.3 | -9.3 |
|  | 2009 | 88.1 | 91.4 | 0.8 | -0.214 | -19.6 | -8.4 |
|  | 2010 | 87.9 | 84.8 | 0.8 | -0.213 | -18.1 | -7.6 |
|  | 2011 | 77.4 | 81.2 | 0.8 | -0.183 | -14.9 | -6.1 |
|  | 2012 | 82.4 | 73.5 | 0.8 | -0.197 | -14.5 | -5.9 |
|  | 2013 | 93.5 | 70.2 | 0.8 | -0.230 | -16.1 | -6.5 |
|  | 2014 | 88.1 | 69.7 | 0.8 | -0.214 | -14.9 | -6.0 |
|  | 2015 | 88.1 | 66.9 | 0.8 | -0.214 | -14.3 | -5.6 |
|  | 2016 | 94.1 | 62.4 | 0.8 | -0.232 | -14.5 | -5.7 |
| Stroke | 1990 | 88.1 | 79.9 | 1.04 | 0.034 | 2.7 | 1.6 |
|  | 1991 | 93.5 | 76.1 | 1.04 | 0.036 | 2.7 | 1.6 |
|  | 1992 | 88.1 | 74.1 | 1.04 | 0.034 | 2.5 | 1.4 |
|  | 1993 | 87 | 72.8 | 1.04 | 0.034 | 2.4 | 1.4 |
|  | 1994 | 88.1 | 73.4 | 1.04 | 0.034 | 2.5 | 1.4 |
|  | 1995 | 87.8 | 70.0 | 1.04 | 0.034 | 2.4 | 1.3 |
|  | 1996 | 88.1 | 68.9 | 1.04 | 0.034 | 2.3 | 1.3 |
|  | 1997 | 88.1 | 54.9 | 1.04 | 0.034 | 1.9 | 1.0 |
|  | 1998 | 90.6 | 52.8 | 1.04 | 0.035 | 1.8 | 1.0 |
|  | 1999 | 88.1 | 51.3 | 1.04 | 0.034 | 1.7 | 0.9 |
|  | 2000 | 88.1 | 48.6 | 1.04 | 0.034 | 1.7 | 0.8 |
|  | 2001 | 82.5 | 45.2 | 1.04 | 0.032 | 1.4 | 0.7 |
|  | 2002 | 88.1 | 45.1 | 1.04 | 0.034 | 1.5 | 0.7 |
|  | 2003 | 88.1 | 44.1 | 1.04 | 0.034 | 1.5 | 0.7 |
|  | 2004 | 90.7 | 41.8 | 1.04 | 0.035 | 1.5 | 0.7 |
|  | 2005 | 88.1 | 37.8 | 1.04 | 0.034 | 1.3 | 0.6 |
|  | 2006 | 88.1 | 37.9 | 1.04 | 0.034 | 1.3 | 0.6 |
|  | 2007 | 89.9 | 37.0 | 1.04 | 0.035 | 1.3 | 0.6 |
|  | 2008 | 88.2 | 36.5 | 1.04 | 0.034 | 1.2 | 0.5 |
|  | 2009 | 88.1 | 33.3 | 1.04 | 0.034 | 1.1 | 0.5 |
|  | 2010 | 87.9 | 32.2 | 1.04 | 0.034 | 1.1 | 0.5 |
|  | 2011 | 77.4 | 33.1 | 1.04 | 0.030 | 1.0 | 0.4 |
|  | 2012 | 82.4 | 30.4 | 1.04 | 0.032 | 1.0 | 0.4 |
|  | 2013 | 93.5 | 28.5 | 1.04 | 0.036 | 1.0 | 0.4 |
|  | 2014 | 88.1 | 28.3 | 1.04 | 0.034 | 1.0 | 0.4 |
|  | 2015 | 88.1 | 27.9 | 1.04 | 0.034 | 0.9 | 0.4 |
|  | 2016 | 94.1 | 26.9 | 1.04 | 0.036 | 1.0 | 0.4 |
| Breast cancer | 1990 | 84 | 31.0 | 1.14 | 0.105 | 3.3 | 1.9 |
|  | 1991 | 82.5 | 31.1 | 1.14 | 0.104 | 3.2 | 1.9 |
|  | 1992 | 82.5 | 29.5 | 1.14 | 0.104 | 3.1 | 1.8 |
|  | 1993 | 82.5 | 31.2 | 1.14 | 0.104 | 3.2 | 1.8 |
|  | 1994 | 82.5 | 30.7 | 1.14 | 0.104 | 3.2 | 1.7 |
|  | 1995 | 82.5 | 29.6 | 1.14 | 0.104 | 3.1 | 1.7 |
|  | 1996 | 82.5 | 28.8 | 1.14 | 0.104 | 3.0 | 1.6 |
|  | 1997 | 82.5 | 28.0 | 1.14 | 0.104 | 2.9 | 1.5 |
|  | 1998 | 88.1 | 26.7 | 1.14 | 0.110 | 2.9 | 1.5 |
|  | 1999 | 82.5 | 25.5 | 1.14 | 0.104 | 2.6 | 1.3 |
|  | 2000 | 85 | 24.8 | 1.14 | 0.106 | 2.6 | 1.3 |
|  | 2001 | 78 | 24.9 | 1.14 | 0.098 | 2.4 | 1.2 |
|  | 2002 | 82.5 | 25.4 | 1.14 | 0.104 | 2.6 | 1.3 |
|  | 2003 | 82.5 | 24.9 | 1.14 | 0.104 | 2.6 | 1.2 |
|  | 2004 | 88.4 | 23.7 | 1.14 | 0.110 | 2.6 | 1.2 |
|  | 2005 | 84 | 23.9 | 1.14 | 0.105 | 2.5 | 1.1 |
|  | 2006 | 82.5 | 22.3 | 1.14 | 0.104 | 2.3 | 1.0 |
|  | 2007 | 87.9 | 22.3 | 1.14 | 0.110 | 2.4 | 1.1 |
|  | 2008 | 74.1 | 22.6 | 1.14 | 0.094 | 2.1 | 0.9 |
|  | 2009 | 82.5 | 22.1 | 1.14 | 0.104 | 2.3 | 1.0 |
|  | 2010 | 85.8 | 21.8 | 1.14 | 0.107 | 2.3 | 1.0 |
|  | 2011 | 77.4 | 21.9 | 1.14 | 0.098 | 2.1 | 0.9 |
|  | 2012 | 77.3 | 20.6 | 1.14 | 0.098 | 2.0 | 0.8 |
|  | 2013 | 82.5 | 20.5 | 1.14 | 0.104 | 2.1 | 0.9 |
|  | 2014 | 86.47 | 19.6 | 1.14 | 0.108 | 2.1 | 0.9 |
|  | 2015 | 82.5 | 20.1 | 1.14 | 0.104 | 2.1 | 0.8 |
|  | 2016 | 83 | 19.9 | 1.14 | 0.104 | 2.1 | 0.8 |
| Bowel cancer | 1990 | 88.1 | 29.7 | 1.21 | 0.156 | 4.6 | 2.7 |
|  | 1991 | 93.5 | 29.5 | 1.21 | 0.164 | 4.8 | 2.9 |
|  | 1992 | 88.1 | 29.4 | 1.21 | 0.156 | 4.6 | 2.6 |
|  | 1993 | 87 | 29.3 | 1.21 | 0.154 | 4.5 | 2.5 |
|  | 1994 | 88.1 | 29.9 | 1.21 | 0.156 | 4.7 | 2.5 |
|  | 1995 | 87.8 | 28.4 | 1.21 | 0.156 | 4.4 | 2.4 |
|  | 1996 | 88.1 | 28.5 | 1.21 | 0.156 | 4.4 | 2.4 |
|  | 1997 | 88.1 | 28.2 | 1.21 | 0.156 | 4.4 | 2.3 |
|  | 1998 | 90.6 | 27.2 | 1.21 | 0.160 | 4.3 | 2.2 |
|  | 1999 | 88.1 | 26.6 | 1.21 | 0.156 | 4.2 | 2.1 |
|  | 2000 | 88.1 | 26.3 | 1.21 | 0.156 | 4.1 | 2.1 |
|  | 2001 | 82.5 | 25.7 | 1.21 | 0.148 | 3.8 | 1.9 |
|  | 2002 | 88.1 | 24.7 | 1.21 | 0.156 | 3.9 | 1.9 |
|  | 2003 | 88.1 | 24.7 | 1.21 | 0.156 | 3.9 | 1.9 |
|  | 2004 | 90.7 | 23.4 | 1.21 | 0.160 | 3.8 | 1.7 |
|  | 2005 | 88.1 | 23.4 | 1.21 | 0.156 | 3.6 | 1.6 |
|  | 2006 | 88.1 | 22.6 | 1.21 | 0.156 | 3.5 | 1.6 |
|  | 2007 | 89.9 | 22.1 | 1.21 | 0.159 | 3.5 | 1.5 |
|  | 2008 | 88.2 | 22.2 | 1.21 | 0.156 | 3.5 | 1.5 |
|  | 2009 | 88.1 | 22.0 | 1.21 | 0.156 | 3.4 | 1.5 |
|  | 2010 | 87.9 | 21.1 | 1.21 | 0.156 | 3.3 | 1.4 |
|  | 2011 | 77.4 | 20.2 | 1.21 | 0.140 | 2.8 | 1.2 |
|  | 2012 | 82.4 | 20.0 | 1.21 | 0.148 | 2.9 | 1.2 |
|  | 2013 | 93.5 | 19.8 | 1.21 | 0.164 | 3.2 | 1.3 |
|  | 2014 | 88.1 | 19.1 | 1.21 | 0.156 | 3.0 | 1.2 |
|  | 2015 | 88.1 | 19.2 | 1.21 | 0.156 | 3.0 | 1.2 |
|  | 2016 | 94.1 | 18.8 | 1.21 | 0.165 | 3.1 | 1.2 |
| Injury | 1990 | 88.1 | 15.5 | 13 | 0.914 | 14.1 | 8.4 |
|  | 1991 | 93.5 | 13.5 | 13 | 0.918 | 12.4 | 7.4 |
|  | 1992 | 88.1 | 12.6 | 13 | 0.914 | 11.5 | 6.6 |
|  | 1993 | 87 | 11.7 | 13 | 0.913 | 10.7 | 6.0 |
|  | 1994 | 88.1 | 11.7 | 13 | 0.914 | 10.6 | 5.8 |
|  | 1995 | 87.8 | 12.0 | 13 | 0.913 | 11.0 | 6.0 |
|  | 1996 | 88.1 | 11.3 | 13 | 0.914 | 10.3 | 5.6 |
|  | 1997 | 88.1 | 10.5 | 13 | 0.914 | 9.6 | 5.1 |
|  | 1998 | 90.6 | 10.1 | 13 | 0.916 | 9.3 | 4.8 |
|  | 1999 | 88.1 | 10.1 | 13 | 0.914 | 9.2 | 4.7 |
|  | 2000 | 88.1 | 10.1 | 13 | 0.914 | 9.2 | 4.7 |
|  | 2001 | 82.5 | 9.8 | 13 | 0.908 | 8.9 | 4.5 |
|  | 2002 | 88.1 | 9.4 | 13 | 0.914 | 8.6 | 4.2 |
|  | 2003 | 88.1 | 8.6 | 13 | 0.914 | 7.9 | 3.8 |
|  | 2004 | 90.7 | 8.0 | 13 | 0.916 | 7.3 | 3.4 |
|  | 2005 | 88.1 | 7.8 | 13 | 0.914 | 7.1 | 3.2 |
|  | 2006 | 88.1 | 8.3 | 13 | 0.914 | 7.6 | 3.4 |
|  | 2007 | 89.9 | 7.8 | 13 | 0.915 | 7.1 | 3.1 |
|  | 2008 | 88.2 | 7.1 | 13 | 0.914 | 6.5 | 2.8 |
|  | 2009 | 88.1 | 7.2 | 13 | 0.914 | 6.6 | 2.9 |
|  | 2010 | 87.9 | 6.8 | 13 | 0.913 | 6.2 | 2.6 |
|  | 2011 | 77.4 | 6.2 | 13 | 0.903 | 5.6 | 2.3 |
|  | 2012 | 82.4 | 6.0 | 13 | 0.908 | 5.5 | 2.2 |
|  | 2013 | 93.5 | 5.7 | 13 | 0.918 | 5.2 | 2.1 |
|  | 2014 | 88.1 | 5.5 | 13 | 0.914 | 5.0 | 2.0 |
|  | 2015 | 88.1 | 5.4 | 13 | 0.914 | 5.0 | 1.9 |
|  | 2016 | 94.1 | 5.6 | 13 | 0.919 | 5.1 | 2.0 |

### PR_d_= prevalence of drinking; Mortality= age standardized mortality; RR= relative risk; PAF= population attributable fraction; PAR _alcohol_ = alcohol attributable burden of death; PAR _beer_ = beer attributable burden of death

### REFERENCES

1. AIHW. Australian Institute of Health and Welfare (AIHW): Health conditions, disability & deaths Canberra 2020 [Available from: <https://www.aihw.gov.au/reports-data/health-conditions-disability-deaths>.

2. Max G, Nancy F, Caitlin H, et al. Alcohol use and burden for 195 countries and territories, 1990-2016: a systematic analysis for the Global Burden of Disease Study 2016. *Lancet* 2018;392(10152):1015-35. doi: 10.1016/s0140-6736(18)31310-2 [published Online First: 2018/08/28]

3. ABS. Australian Bureau of Statistics. 3412.0 Migration, Australia, 2007–08. , 2009.

4. IBISWorld. Liquor Retailing in Australia 2020 [Available from: <https://my.ibisworld.com/>.

5. AIHW. Alcohol, tobacco & other drugs in Australia 2020 [Available from: <https://www.aihw.gov.au/reports/alcohol/alcohol-tobacco-other-drugs-australia/contents/drug-types/alcohol>.

6. Zhao J, Stockwell T, Roemer A, et al. Alcohol Consumption and Mortality From Coronary Heart Disease: An Updated Meta-Analysis of Cohort Studies. *Journal of studies on alcohol and drugs* 2017;78(3):375-86. doi: 10.15288/jsad.2017.78.375 [published Online First: 2017/05/13]

7. Diem P, Deplazes M, Fajfr R, et al. Effects of alcohol consumption on mortality in patients with Type 2 diabetes mellitus. *Diabetologia* 2003;46(11):1581-85. doi: 10.1007/s00125-003-1209-2

8. Patra J, Taylor B, Irving H, et al. Alcohol consumption and the risk of morbidity and mortality for different stroke types--a systematic review and meta-analysis. *BMC public health* 2010;10:258. doi: 10.1186/1471-2458-10-258 [published Online First: 2010/05/21]

9. Yun-Jiu G, Ding-Xiong X, Ke-Hu Y, et al. Alcohol Consumption and Breast Cancer Survival: A Metaanalysis of Cohort Studies. *Asian Pacific Journal of Cancer Prevention* 2013;14(8):4785-90.

10. Wang Y, Duan H, Yang H, et al. A pooled analysis of alcohol intake and colorectal cancer. *Int J Clin Exp Med* 2015;8(5):6878-89.

11. Taylor B, Rehm J. The relationship between alcohol consumption and fatal motor vehicle injury: high risk at low alcohol levels. *Alcoholism, clinical and experimental research* 2012;36(10):1827-34. doi: 10.1111/j.1530-0277.2012.01785.x [published Online First: 2012/05/07]
